# Postprocessing for Skin Detection

**DOI:** 10.3390/jimaging7060095

**Published:** 2021-06-03

**Authors:** Diego Baldissera, Loris Nanni, Sheryl Brahnam, Alessandra Lumini

**Affiliations:** 1 Department of Information Engineering (DEI), University of Padova, 35131 Padova, Italy; loris.nanni@unipd.it; 2 Department of Information Technology and Cybersecurity, Missouri State University, Springfield, MO 65804, USA; sbrahnam@missouristate.edu; 3 Department of Computer Science and Engineering (DISI), University of Bologna, 47521 Cesena, Italy; alessandra.lumini@unibo.it

**Keywords:** segmentation, skin detector, convolutional neural networks, postprocessing

## Abstract

Skin detectors play a crucial role in many applications: face localization, person tracking, objectionable content screening, etc. Skin detection is a complicated process that involves not only the development of apposite classifiers but also many ancillary methods, including techniques for data preprocessing and postprocessing. In this paper, a new postprocessing method is described that learns to select whether an image needs the application of various morphological sequences or a homogeneity function. The type of postprocessing method selected is learned based on categorizing the image into one of eleven predetermined classes. The novel postprocessing method presented here is evaluated on ten datasets recommended for fair comparisons that represent many skin detection applications. The results show that the new approach enhances the performance of the base classifiers and previous works based only on learning the most appropriate morphological sequences.

## 1. Introduction

A highly relevant segmentation problem is skin detection, a problem that discriminates regions in images and videos into the two classes skin and nonskin. The applications of skin detection are many, as are the challenges. Skin detection is a valuable component in locating faces, tracking individuals, human–computer interactions, biometric authentication, medical imaging, and objectionable content screening [1]. Challenges are concerned not only with building powerful classifiers but also with developing all the additional methods required to accomplish the task, including data preprocessing and postprocessing.

When discussing the history of a machine learning problem, it is often appropriate to divide developments into two periods: those before and those after the rise of deep learning. Prior to deep learning, most human skin detection approaches were based either on skin color separation or texture features. Skin color separation assumes that skin tones can be distinguished from background colors via a clustering rule in a color space [2]. A survey of the literature comparing different color spaces is available in reference [3]. The authors in that review surveyed four types of color models: (1) basic color models like RGB and normalized RGB, (2) perceptual models like HIS and HSV, (3) perceptual uniform models like CIE-Lab and CIE-Luv, and (4) orthogonal models like YCbCr and YIQ. A more challenging way to tackle skin detection is to assume a nonconstrained environment [3]; this type of approach involves training classifiers that have the capacity to generalize. Some skin segmentation examples along this line include using a multilayer perceptron [4], random forest [5], Bayesian classifiers [6], and adaptive discriminative analysis [7]. Another class of early approaches relies on image segmentation, where pixel neighborhoods are examined to segment regions where human skin is located [8,9].

Developments in skin detection in the deep learning age often involve applying Convolutional Neural Networks (CNN) to skin segmentation. CNNs are well-known for their superior performance in image classification and in segmentation tasks involving pixel-based prediction like scene labeling. One of the first works to apply deep learning to human skin detection was reference [10], where patch-based networks trained on a dataset of patches were shown to compete well with pixel-wise classification. Later, in reference [11], a procedure for human skin detection was designed that combined recurrent neural network (RNN) layers with fully CNN (FCN). The advantage of this combination was that the FCN layers captured local features while the RNN layers modeled the semantic contextual dependencies. In reference [12], an inception-based architecture was proposed that was composed of convolutional and inception modules with training considering both patches and whole images. In reference [13], the authors performed experiments using several CNN architectures and concluded that DeepLabv3+ is the best CNN for skin segmentation. Two very recent skin segmentation architectures are OR-Skip-Net [14], a model that transfers direct edge information across the network in such a way as to empower the features, and Skinny [15], based on a lightweight U-Net. A deep learning approach based on transfer learning was proposed in reference [16] for skin detection in gray-scale images; this model is based on the combination of a colorization network and a pretrained Inception-ResNet-v2.

Several studies have been conducted that provide comprehensive evaluations of CNN architectures with the intention of finding the most promising ones [13,17,18]. For example, in reference [17], an extensive evaluation was conducted that compared the best pixel-based methods with CNN approaches both in-domain and cross-domain. In reference [18], experiments were performed on many different CNN structures in an attempt to isolate those that work best for skin detection. Besides providing an exhaustive comparison of skin color detection approaches, the authors in reference [13] developed a framework for fair comparisons of methods in skin detection. The advantages of using deep learning methods for skin detection (as compared with pixel-based and classical region-based approaches) were demonstrated in the experiments performed in references [13,17].

The results from comprehensive evaluations of both CNN architectures and handcrafted methods have revealed much room for performance improvement, mainly when it comes to low-quality input images where postprocessing is often required to improve the segmentation performance. In the literature, several postprocessing ideas have been proposed to improve the performance of skin segmentation. In reference [19], for example, a simple rejection rule was developed to remove small noisy regions from the final mask, while, in reference [20], an adaptive method based on a blob analysis was proposed to improve the classification performance of color-based skin detectors. The methods designed for pixel-based segmentation approaches, which classify every pixel separately, can produce high false positives for background pixels when the color is too similar to human skin. Deep learning approaches do not suffer from these problems since they are based on a global analysis of the image. A recent work [21] has suggested the application of learned morphological operators to improve the performance of deep learners in skin detection. In learned postprocessing, a classifier learns the best postprocessing method to use based on the type of image under consideration.

In this paper, a new learned postprocessing method for skin detection is proposed that applies either a set of morphological processes or a homogeneity function. Mathematical morphologies provides powerful nonlinear operators for a variety of image processing tasks, such as filtering, segmentation, and edge detection. In some recent works, novel morphological layers have been proposed to include learned morphological operators inside a deep learning framework, mainly for applications of image classification, denoising, edge detection, or super-resolution [22,23]. In this study, as in reference [21], which this work extends, skin is detected, first, by segmenting the image with a deep learner (or another handcrafted approach) and, second, by applying a postprocessing method. This approach involves classifying an image into several classes (predetermined by the researchers) that govern which postprocessing method to apply. Therefore, in this work, the postprocessing step is based on handcrafted processes, while a learning procedure is used to select the appropriate approach. In reference [21], five image classes were identified for using different sets of morphological processes. Here, eleven classes have been identified. The collection of possible postprocessing methods includes not only the morphological processes but, also, a homogeneity function. The main aim of this work is to find a criterion for selecting images that need a homogeneity postprocessing method instead of a morphological approach.

Following the testing framework developed in reference [13], the performance results of the learning procedure proposed here to select the appropriate postprocessing functions (either morphological or homogeneity) are compared to several state-of-the-art detectors without postprocessing and to the results produced by the postprocessing methods proposed in reference [21]. A series of experiments demonstrate that the novel postprocessing method improves the detection across several datasets representing different applications and is superior to that introduced in reference [21]. Additionally, several training protocols are examined that show the usefulness of a learned postprocessing approach, even without specific training on the datasets.

The remainder of this paper is organized as follows. In Section 2, the general approach for learning the best method to take in the postprocessing phase is described. This section includes a discussion of CNN fine-tuning for skin detection. In Section 3, the testing framework for comparing skin detection systems is described, and a set of experiments are presented that demonstrate the ability of the proposed postprocessing method to boost the performance of state-of-the-art skin detectors. This paper concludes in Section 4 by offering some directions for further research.

## 2. System Description

### 2.1. Skin Detection with Deep Learners

The application of deep learning to the general problem of segmentation is relatively recent [24], and, as happened in other applications, it immediately outperformed the previous state-of-the-art approaches based on handcrafted methods. Long et al. [25] were among the first to adapt deep learners (AlexNet and GoogLeNet) to the task of segmentation by fine-tuning the FCN layers of these deep learners. Another powerful deep learner tailored for image segmentation is the encoding/decoding network SegNet [26]. The encoder part of SegNet is the same as the convolutional layers in the VGG16 network [27]. In SegNet, each of the thirteen encoders is coupled with a decoder. As weights can be trained for classification on larger datasets, the FCN layers are discarded in favor of high-resolution feature maps at the deepest encoder output. Yet another deep learner for segmentation is the U-shaped network [28]; in this network, the task of the encoder is to classify the object, and the task of the decoder is to demarcate the pixel position. Since deep learners require large numbers of annotated training samples, pretrained deep architectures are used for the encoding component; the network is then fine-tuned for the specific problem.

As in [29], the deep learner for image segmentation is Deeplabv3+ [30]. Its predecessor, Deeplabv3 [31], uses atrous convolution [32], or dilated convolution, to repurpose pretrained networks in order to control the resolution of feature responses without adding extra parameters. To handle multiple scales, Deeplabv3 also applies spatial pyramid pooling to capture the context at several ranges. Deeplabv3+ adds a simple decoder module to the original architecture that refines the segmentation results along the object boundaries.

The encoder–decoder structure of DeepLabv3+ allows it to be built on top of a pretrained CNN, such ResNet50, the architecture used here (internal preliminary investigations showed that both ResNet101 and ResNet34 produced similar results to ResNet50). Following the methodology outlined in reference [29], all models for skin segmentation were trained on a dataset containing 2000 images using class weighting; the training parameters used in this work, however, are the following: batch size (30), learning rate (0.001), and max epoch (50), with data augmentation set to 30 epochs.

For comparison purposes, an ensemble of classifiers is designed to fuse three variants of DeepLabv3+ with the variants constructed by substituting all the activation layers with a different activation function, as in reference [29], so that the diversity of the networks can improve the ensemble performance. According to the experiments in [29], which investigated many different activation functions using this approach, one of the best-performing ensembles for skin detection is FusAct3, a lightweight ensemble obtained by the fusion of DeepLabv3+ on three variants of the standard ReLU function: MeLU, wMeLU, and PReLU [33]. Since DeepLabv3+ and the ensemble FusAct3 gain a state-of-the-art performance for this problem, we used these approaches as a baseline of this research. Even though, in theory, the proposed postprocessing approach can be coupled with any skin segmentation method, including pixel-based approaches or adaptive methods, the learning processes and all studies here were carried out starting from the masks obtained from the deep learners.

### 2.2. Postprocessing

**Figure 1:**
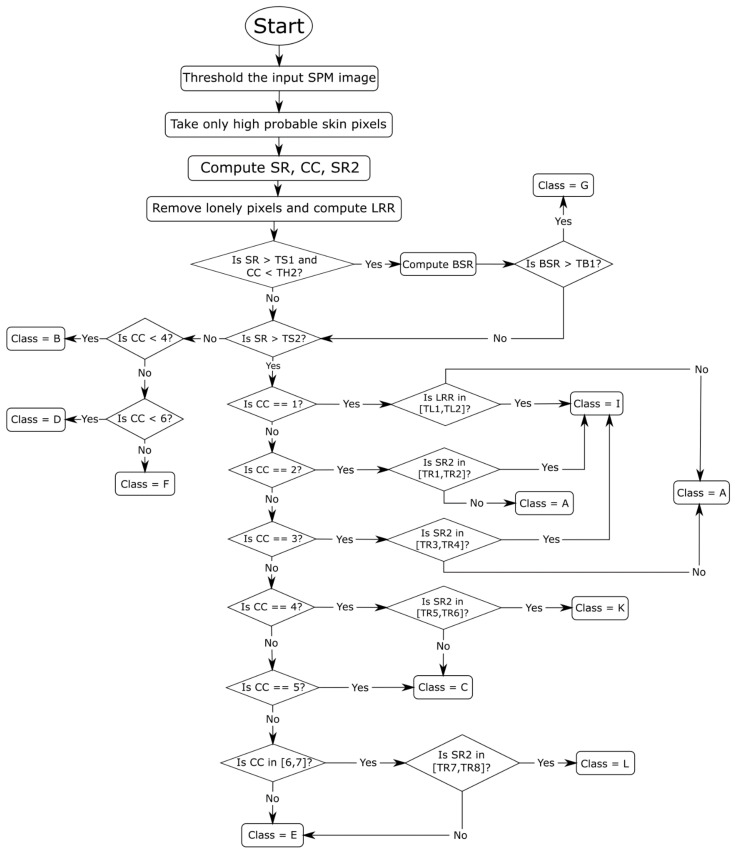
Flow chart of the image classification approach.

In this paper, we expanded the approach taken in reference [21] for refining the segmentation results of a CNN applied to skin detection, an approach that is based on learning to identify classes of images that will then govern the selection of the morphological postprocessing method. Unlike the method proposed in reference [21], the detected classes in this work determine two types of postprocessing techniques: a set of morphological functions and a homogeneity function. As was the case in reference [21], the classes were determined by a visual inspection of images. After that, a learning procedure was designed to automatically classify each image into one of several classes according to the flow chart in Figure 1.

As mentioned in the introduction, five such classes were originally identified in reference [21]; in this work, a postprocessing method was chosen (see Table 1) based on one of the following eleven classes:

- Class A: images in which there is a very near foreground that implies a high percentage of skin pixels and few connected components.
- Class B: images in which there is only the face of the person that is not too close to the camera.
- Class C: images where there is a single person with body parts showing, e.g., the face, arms, and legs, on separated connected components.
- Class D: images in which there is only one person far from the camera.
- Class E: images in which there is a group of people and no connected component that prevails over the others.
- Class F: images in which there is the presence of a far group of people, which implies an elevated number of connected components and a few skin pixels with respect to the total number of image pixels.
- Class G: images in which the background is erroneously classified as skin pixels.
- Class H: images without skin.
- Class I: images in which the foreground is very close, and the largest connected component takes almost all the skin pixels.
- Class K: images in which there is a single person and a connected component that, regarding dimensions, overcome the others.
- Class L: images in which there is a small group of people and a connected component that triumphs over the others.

After the class assignment, the selected postprocessing method was applied to create the final mask.

Compared to the classes identified in reference [21], classes B–D are expanded into several categories. Classes C and D, originally having to do with groups, are further broken down here into classes E, F, and L, and class B (originally called “Face in foreground”) is generalized here into classes D and K to include all the body parts. Moreover, the notion of the foreground is further expanded into classes A, B, and I to distinguish different foreground situations. It should also be pointed out that the only difference between classes A, B, and C and classes I, K, and L in Table 1 is the type of postprocessing method applied in the final step.

A CNN applied to skin detection provides an image called the Skin Probability Map (SPM), which corresponds to the classifier score at the pixel level, i.e., the probability of each pixel to be labeled as skin. This probability was rescaled to the range [0, 255] and taken as the input of our postprocessing method. Binary classification was performed on every pixel to the classes skin/nonskin based on the postprocessing function. The SPM was passed into a threshold algorithm based on Otsu’s method [34], where an optimal threshold was selected by the discriminant criterion. In this case, the pixels were classified as belonging to one of three sets: a high, discrete, or low probability of being a skin pixel, depending on the pixel’s value. Only pixels belonging to the first set were considered true skin.

In reference [21], three features (items 1–3 below) were computed to assign the image to the right class. The method proposed here includes two additional features (items 4 and 5):

(1) Skin Ratio (SR) [2]: the percentage of skin pixels in the image;
(2) Connected Components (CC) [2]: the number of skin regions;
(3) Border Skin Ratio (BSR) [2]: a measurement reflecting the amount of skin surface recognized within a 1-pixel frame as a border. It is the ratio between the numbers of pixels of skin detected on the top, right, and left sides (excluding the bottom) of the image over the total number of pixels in those sides;
(4) SR2: similar to SR, except that the ratio between the largest skin region area and the number of skin pixels is computed;
(5) Largest Region Ratio (LRR): the ratio between the largest region area and the image dimension; LRR is useful only when the image has a foreground that is near. After computing this value, all skin regions composed of only one pixel are removed.

Depending on the assumed values of these features, the image is assigned to one of the eleven possible classes, as shown in Figure 1 and in the pseudocode below.

There are four possible postprocessing methods, three based on different sets of morphological operators, such as multiplication and dilatation, and one on homogeneity [3], a region-based algorithm. The homogeneity approach checks the pixel and its neighborhood before classifying it in the following way:

(1) The SPM image is normalized; and, only in the first loop, a threshold *T* is set to 0.2, so that if a pixel has a value greater than *T* it will be labeled as skin;
(2) Regions with an area less than 300 pixels are removed;
(3) Every region is evaluated to decide whether it can be homogenous or not using this formula:

$$\left(\sigma <45\right)\;AND\;\left(\left(\frac{N_{e}}{N_{d}}\leq 3.5\right)OR\left(\frac{N_{e}}{N_{s}}\leq 0.02\right)\right),$$

where σ is the standard deviation of the skin pixel in the region, $N_{e}$ is the number of the border pixels (computed with the Sobel operator), $N_{s}$ is the number of skin pixels, and $N_{d}$ is the maximum size of the region’s bounding box. If all the regions are homogenous, the actual mask will be returned; otherwise, *T* will be increased by 10%, and the algorithm will return to the first step.

It has been demonstrated that the homogeneity method works well when people are near [3]. Therefore, the values of CC, SR2, and LRR are used to select the appropriate images to be processed by this algorithm (Algorithm 1).

The other postprocessing functions are based on the five morphological operators (see Table 2) used in reference [21] but which are combined here in three sequences (see Table 3).

**Algorithm 1** Pseudocode of the learned postprocessing method.
1: postP(spm)
2:  class ← H
3:  img ← threshold(spm)
4:  compute SR, CC, SR2 on img
5:  imgO2 ← remove lonely pixels
6:  compute LRR on imgO2
7:  if SR > TS1 & CC < 6
8:   compute BSR on img
9:   if BSR > TB1
10:    class ← G
11:  if (SR > TS2) & (class NOT G)
12:   if CC == 1
13:    if LRR in [TL1, TL2]
14:      class ← I
15:    else
16:       class ← A
17:   if CC == 2
18:    if SR2 in [TR1, TR2]
19:       class ← I
20:    else
21:       class ← A
22:   if CC == 3
23:    if SR2 in [TR3, TR4]
24:       class ← I
25:    else
26:       class ← A
27:   if CC == 4
28:    if SR2 in [TR5, TR6]
29:       class ← K
30:    else
31:       class ← C
32:   if CC == 5
33:      class ← C
34:   if CC in [6,7]
35:    if SR2 in [TR7, TR8]
36:       class ← L
37:    else
38:       class ← E
49:   else
40:      class ← E
41:  if (SR < TS3) & (class NOT G)
42:   if CC < 4
43:      class ← B
44:   if CC < 6
45:      class ← D
46:   else
47:      class ← F
48:  switch class
49:   case I, K, L
50:    mask ← homogeneity(img)
51:   case A, B, C, E
52:    mask ← morphological_set#1
53:   case D, F
54:    mask ← morphological_set#2
55:   case G
56:    mask ← morphological_set#3
57:  return mask

The value of all the thresholds (see Table 4) used in the algorithm above are learned from the training set of the ECU dataset.

## 3. Experimental Results

Making fair comparisons between the skin detection algorithms and datasets is problematic, because many datasets are created by different research groups, have unreliable ground truths (mislabeled sections), and are intended for many separate applications. For fair comparisons, the authors of reference [13] proposed a framework for skin detector comparisons that is based on how well new algorithms perform across the following ten datasets:

(1) ECU [36]: a dataset that contains 4000 color images. The dataset is divided into two, with the first half making up the training set;
(2) Compaq [37]: a widely used skin dataset that contains 4675 images;
(3) UChile [38]: a challenging, though relatively small dataset, that includes 103 images with complex backgrounds and different illumination conditions;
(4) Schmugge [39]: 845 images extracted from several face datasets;
(5) Feeval [40]: a dataset containing 8991 frames that were taken from twenty-five videos of low-quality found online. The performance is the average result considering all the frames for a given video; in other words, each video is viewed as one image when it comes to reporting the performance;
(6) MCG [39]: a dataset containing 1000 images;
(7) VMD [41]: a dataset that includes 285 images collected from a number of different datasets that are publicly available for human action recognition;
(8) SFA [42]: a dataset that contains 3354 skin samples and 5590 nonskin samples extracted from two popular face recognition datasets: the FERET database and the AR Face Database;
(9) Pratheepan [43]: a dataset with only 78 images;
(10) HGR [44]: a dataset intended to evaluate gesture recognition. In this dataset, the image sizes are large. For this reason, the images in HGR2A (85 images) and in HGR2B (574 images) were reduced by 0.3.

For a more extensive description of the ten datasets, see reference [13].

Since skin detection is a binary problem, all standard measures of performance evaluation (accuracy, precision, recall, Kapp, $F_{1}$, and the ROC curve) are appropriate. $F_{1}$ is the most common performance indicator used in the literature, however. For this reason, this indicator is the one recommended by the authors of reference [13] and is defined as

$$F_{1}=2tp/\left(2tp+fn+fp\right)$$

where $F_{1}\in \left[0,1\right]$, fn are false negatives, tp are true positives, and fp are false positives. When used as the performance measure in skin detection, $F_{1}$ is averaged at the pixel level rather than at the image level. This way of averaging is independent of variations in the sizes of images, which are different for each dataset.

The first set of experiments, reported in Table 5 compares the performance for the skin detector DeepLabV3+ and FusAct3 with three different postprocessing methods:

(1) OR: the original method without postprocessing;
(2) MorphBASE [21]: applies morphological operators;
(3) LearnMorph [21]: our previous learned postprocessing method;
(4) MorphHom: the novel approach proposed here.

The Wilcoxon signed-rank test shows that the postprocessing approach proposed here coupled with DeepLabV3+ outperforms all the other methods based on DeepLabV3+ (including OR) with a *p*-value of 0.0001; when coupled with FusAct3, the new postprocessing approach outperforms the other methods based on FusAct3 (including OR) with a *p*-value of 0.1. For both DeepLabV3+ and FusAct3 (see the discussion in Section 2.1), there was no statistical difference between them using OR, MorphBASE, and LearnMorph.

Comparing the stand-alone approaches in Table 5 and the Wilcoxon signed-rank tests, the following can be concluded:

- The quality of the images has an impact on the performance; thus, the performance diverges considerably between datasets.
- When it comes to deep learning, fine-tuning on each dataset is critical. Take, for example, the HGR dataset created for the task of gesture recognition; certainly, segmentation by a CNN specifically trained for this task would produce better results, but obtaining the best performance on a particular dataset is not the aim here. For fair comparisons across all datasets representing different tasks, the same configuration has to be maintained.
- The new postprocessing method improves the performance of the base methods in nearly all the other datasets. This is of particular interest, given that FusAct3 obtains a state-of-the-art performance.

**Figure 2:**
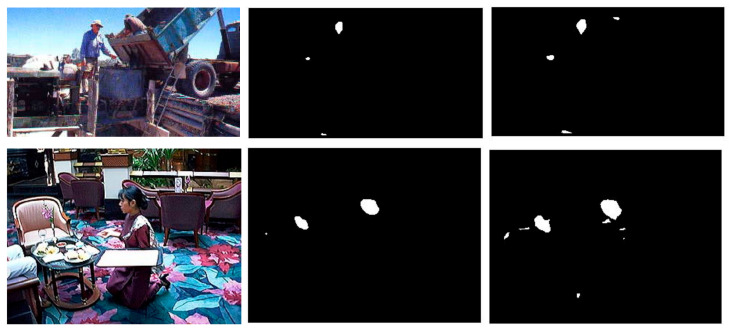

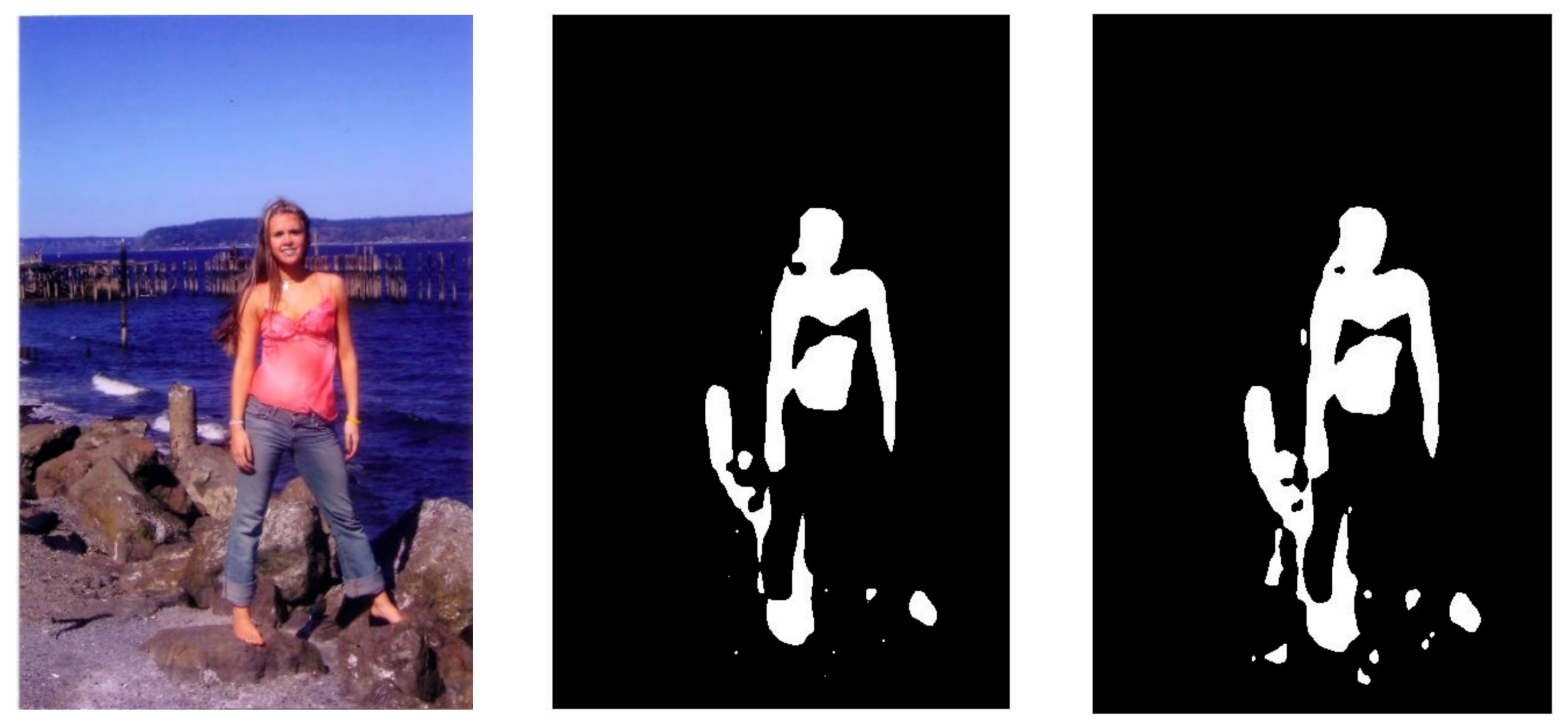
RGB image (**left**), skin mask via FusAct3 + MorphHom (**middle**), and skin mask via FusAct3 (**right**). Sample images in column 1 were taken from the publicly available ECU dataset located at https://documents.uow.edu.au/~phung/download.html, (accessed on 31 May 2021).

Visually examining some of the postprocessing images obtained by the refinement of our new method MorphHom would provide insight into why the performance is improved using it. In Figure 2, three such images are provided with and without the addition of MorphHom.

**Figure 3:**
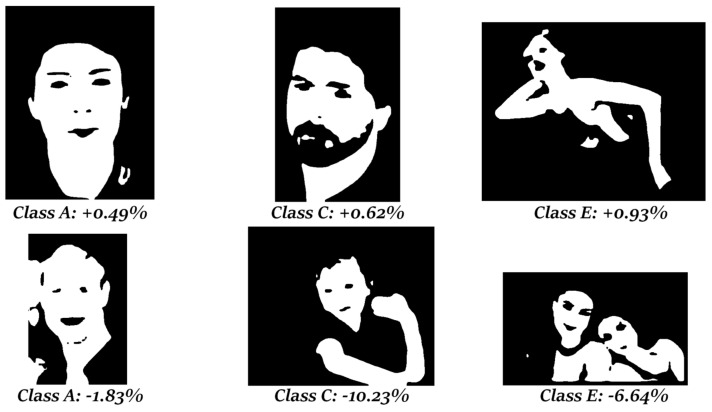
First row: images from the classes (A, C, and E) in which the homogeneity method obtained better results and the relative increment over the F1-measure. Second row: pictures from the same classes in which the homogeneity algorithm gave poor results with respect to the morphological approach.

As noted in the introduction, the main aim of this work compared to [21] is to find a criterion for selecting images in need of a homogeneity postprocessing method instead of a morphological approach. Given the results produced above, it is clear that the homogeneity algorithm improves the final segmentation when there is a connected component that prevails over the others. Some examples and the relative increment/decrement in performance after applying the homogeneity are shown in the following masks (Figure 3).

## 4. Conclusions

A new post-processing method for enhancing the classification of skin detectors was presented in this work that assigns a class to an image and then applies either a series of morphological operators or a homogeneity function to generate the final mask. Eleven classes of images were specified, and the postprocessing method was selected based on the class to which the image belongs. Experimentation across ten datasets representative of several problem domains demonstrated that the application of this approach for learning to select the best postprocessing method from this set of postprocessing possibilities significantly improved the performance of the base skin detectors.

Further studies are planned that will investigate the best postprocessing techniques for low-quality images, since these images failed to perform well with DeepLabV3+. This degradation in performance with low-quality images was probably the consequence of training the deep network on high-quality images only. One way to tackle this problem is to retrain the CNN by applying different types of data augmentation that produce low-quality images and then test whether this improves the segmentation performance on these types of images.

Another interesting topic is the application of morphological operators in the segmentation process. Recently, a few studies [22,23,45] have emerged based on the definition of a new topology of networks, including morphological operators. These semi-hybrid versions of traditional convolution and pseudo-morphological operations could be adapted to the segmentation problem with the aim of substituting the handcrafted postprocessing step. Additional studies should also be conducted to handle the skin segmentation of people of different races using a dataset developed for this purpose, such as that in presented in reference [14], which should be available to the public shortly.

## Figures and Tables

**Table 1:** Image classification classes and the related postprocessing methods.

Class	Short Description	Postprocessing
A	Foreground near	Morphological (Set#1)
B	Foreground far	Morphological (Set#1)
C	With body parts near	Morphological (Set#1)
D	With body parts far	Morphological (Set#2)
E	Group near	Morphological (Set#1)
F	Group far	Morphological (Set#2)
G	Background considered as skin	Morphological (Set#3)
H	No skin	-
I	Foreground near	homogeneity
K	With body parts near	homogeneity
L	Group near	homogeneity

**Table 2:** Morphological MATLAB operators. Note that the parameters I and J are masks, SE is the structuring element and P is a pixel count value.

Morphological Operators	Description
imerode(I, SE)	Erosion
imdilate(I, SE)	Dilation
imfill(I, ‘holes’)	Mask-filling ‘holes’
immultiply(I, J)	Pixel-by-pixel multiplication of the two masks
bwareaopen(I, P)	Area removal from the mask with pixel count < P

**Table 3:** Morphological operations (listed as MATLAB commands [35]) performed on each class, except H (no skin). Note: although the first two rows use the same set of operators, the disks employed as structuring elements have different sizes.

Class	Morphological Set
A, B, C, E	(Set#1) = imerode→bwareaopen→imdilate→immultiply
D, F	(Set#2) = imerode→bwareaopen→imdilate→immultiply
G	(Set#3) = imerode→immultiply→imfill

**Table 4:** Thresholds used in the pseudocode.

TS1 = 0.12	TS2 = 0.1685	TS3 = 0.1685	TB1 = 1	TL1 = 3.5e-6	TL2 = 86.3e-6	TR1 = 0.9967
TR2 = 0.9995	TR3 = 0.9730	TR4 = 0.9992	TR5 = 0.9701	TR6 = 0.9979	TR7 = 0.8736	TR8 = 0.9984

**Table 5:** F1-measure of the tested methods (N.B. bold-face indicates superior results).

Method		DataSet											Avg
		FV	Prat	MCG	UC	CMQ	SFA	HGR	Sch	VMD	ECU	VT	
DeepLabV3+	OR	0.759	0.831	0.872	0.881	0.799	0.946	0.950	0.763	0.592	0.917	0.679	0.817
	MorphBASE	0.763	0.835	0.874	0.882	0.803	0.946	0.951	0.765	0.583	0.918	0.679	0.818
	LearnMorph	0.762	0.835	0.874	0.882	0.788	0.946	0.951	0.766	0.583	0.918	0.679	0.817
	MorphHom	0.765	0.852	0.877	**0.888**	0.813	0.947	0.957	0.773	0.622	0.926	0.691	0.828
FusAct3	OR	0.792	0.854	0.881	0.886	0.817	0.952	0.959	0.771	0.661	0.926	0.710	0.837
	MorphBASE	**0.794**	0.856	**0.882**	0.885	0.819	0.952	0.959	0.771	0.636	0.927	0.710	0.836
	LearnMorph	**0.794**	0.856	**0.882**	0.885	0.807	**0.953**	0.959	0.771	0.636	0.927	0.710	0.835
	MorphHom	0.782	**0.874**	**0.882**	0.885	**0.828**	0.952	**0.963**	**0.774**	**0.669**	**0.935**	**0.717**	**0.842**

## Data Availability

All ten datasets are either publicly available or available upon request by the authors of the papers cited for each dataset. All MATLAB source code is available at https://github.com/LorisNanni/Postprocessing-for-Skin-Detection.
